# Malaria parasite clearance

**DOI:** 10.1186/s12936-017-1731-1

**Published:** 2017-02-23

**Authors:** Nicholas J. White

**Affiliations:** Mahidol Oxford Tropical Medicine Research Unit, Faculty of Tropical Medicine, Mahidol University, 420/6 Rajvithi Road, Bangkok, 10400 Thailand

## Abstract

Following anti-malarial drug treatment asexual malaria parasite killing and clearance appear to be first order processes. Damaged malaria parasites in circulating erythrocytes are removed from the circulation mainly by the spleen. Splenic clearance functions increase markedly in acute malaria. Either the entire infected erythrocytes are removed because of their reduced deformability or increased antibody binding or, for the artemisinins which act on young ring stage parasites, splenic pitting of drug-damaged parasites is an important mechanism of clearance. The once-infected erythrocytes returned to the circulation have shortened survival. This contributes to post-artesunate haemolysis that may follow recovery in non-immune hyperparasitaemic patients. As the parasites mature *Plasmodium vivax*-infected erythrocytes become more deformable, whereas *Plasmodium falciparum*-infected erythrocytes become less deformable, but they escape splenic filtration by sequestering in venules and capillaries. Sequestered parasites are killed in situ by anti-malarial drugs and then disintegrate to be cleared by phagocytic leukocytes. After treatment with artemisinin derivatives some asexual parasites become temporarily dormant within their infected erythrocytes, and these may regrow after anti-malarial drug concentrations decline. Artemisinin resistance in *P. falciparum* reflects reduced ring stage susceptibility and manifests as slow parasite clearance. This is best assessed from the slope of the log-linear phase of parasitaemia reduction and is commonly measured as a parasite clearance half-life. Pharmacokinetic-pharmacodynamic modelling of anti-malarial drug effects on parasite clearance has proved useful in predicting therapeutic responses and in dose-optimization.

## Background

Malaria harms the infected host as a consequence of the blood stage infection. Illness results from the host responses to this infection and the increased destruction of both infected and uninfected erythrocytes. Vital organ pathology in the potentially lethal *Plasmodium falciparum* and *Plasmodium knowlesi* malarias results from microvascular dysfunction [1]. As *P. falciparum* matures the infected erythrocytes adhere to microvascular endothelium (cytoadherence) interfering with vascular function and, at high densities, reducing perfusion. The degree of sequestration and the vital organs affected determine the clinical pattern and outcome of severe falciparum malaria [1, 2]. Cytoadherence is not prominent in the other human malaria parasites.

Anti-malarial drugs damage and eventually kill malaria parasites. This limits the infection and its pathological consequences. The changes in parasite density that occur following anti-malarial treatment can be used to assess the therapeutic response to anti-malarial drugs [3, 4]. Recent developments in ultrasensitive DNA or RNA detection (uPCR) have revealed the previously unseen dynamics of malaria parasite clearance at low densities, and in treatment failure, regrowth following anti-malarial drug treatment. The mechanisms of malaria parasite clearance, the factors affecting it, and the interpretation of parasite clearance data in anti-malarial drug trials are reviewed here.

### Parasite multiplication in the human host

Malaria infection starts with the inoculation of a small number of sporozoites (median number estimated to be about 10) by a probing female anopheline mosquito. These motile parasites pass to the liver within an hour. Having invaded hepatocytes they then begin a period of rapid asexual multiplication [4, 5], dividing approximately every 8 h until each infected liver cell contains thousands of merozoites. Intrahepatic pre-erythrocytic development can be inhibited by some anti-malarials (antifols, 8-aminoquinolines, atovaquone, KAF 156, DMB 265) and some antibiotics (e.g. azithromycin, tetracyclines). In *Plasmodium vivax* infections and in both species of *P. ovale* malaria a sub-population of sporozoites form dormant liver stages called “hypnozoites” which awaken weeks or months later to cause relapses of malaria [4]. The hypnozoites can be killed only by 8-aminoquinolines of the currently available anti-malarial drugs.

### Asexual parasite multiplication

**Fig. 1:**
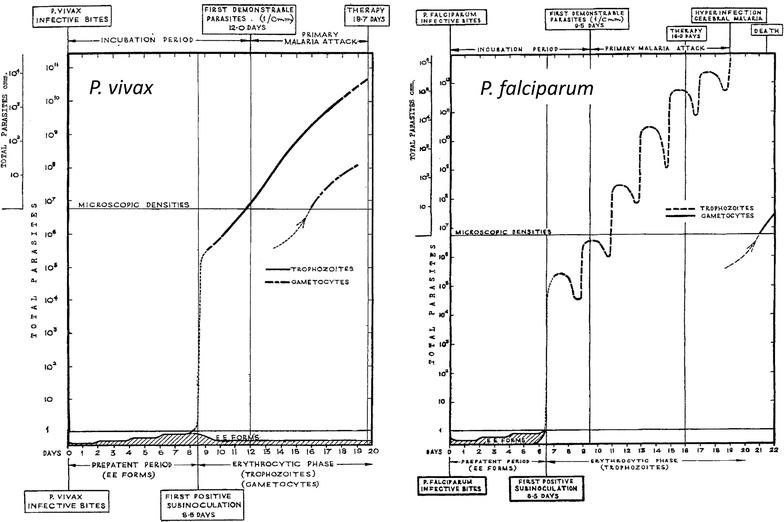
A comparison of parasite dynamics in human malaria infections as illustrated by Fairley [5] following his classic studies of induced malaria in volunteers. The total numbers of parasites in the body of an adult are shown in the *vertical axes*, and time in days is shown in the *horizontal axis*

At the completion of pre-erythrocytic development and following hepatic schizont rupture the newly liberated merozoites enter the blood stream and promptly invade erythrocytes. Then the growing intraerythrocytic malaria parasites begin to consume the red cell contents. The complete life cycle in the red blood cells approximates one day for *P. knowlesi*, two days for *P. falciparum*, *P. vivax* and *Plasmodium ovale* (two species) and three days for *Plasmodium malariae* [4]. A small sub-population of asexual parasites may stop growing and dividing for days or weeks (“dormancy”) [6]. Parasite multiplication rates in non-immune patients in this early stage of infection, before the symptoms of malaria have developed, range typically from 6 to tenfold per cycle (30–50% efficiency), but sometimes reach 20-fold [5, 7–9]. Initial multiplication rates are similar for *P. falciparum* and *P. vivax.* As a result, total parasite numbers in the blood rise exponentially from 10^4^ to 10^5^ in the first asexual cycle to reach 10^8^ after 3–4 cycles (i.e. 6–8 days for *P. falciparum* and *P. vivax*) (Fig. 1). One hundred million parasites in the body of an adult human corresponds with a blood parasite density of about 50/µL [5, 7] and this density is usually associated with the onset of fever and illness in non-immune subjects (a “pyrogenic density”) [10, 11]. The addition of a pre-erythrocytic liver development of 5.5–7 days plus 6–8 days of blood stage multiplication results in the usual incubation period of 11–15 days in falciparum or vivax malaria [10, 11]. People who have had multiple previous malaria infections acquire an antitoxic immunity (“premunition”) which results in higher parasite densities being tolerated without symptoms, although densities over 10,000/µL are usually associated with illness even in areas of high malaria transmission [4, 10–12]. Immunity slows parasite multiplication and accelerates parasite clearance. In most infections after logarithmic parasite multiplication there is an abrupt reduction in parasite multiplication at high densities. Severe malaria is a consequence of a failure of the infecting parasites to stop multiplying [13].

### Sexual stage development

A sub-population of the blood stage parasites commit to sexual development forming male and female gametocytes. This reduces the parasite multiplication rate. Commitment (switching) to sexual development occurs immediately in vivax malaria (which becomes infectious to mosquitoes at, or even below pyrogenic densities) whereas gametocytogenesis is delayed in falciparum malaria (Fig. 1) [14]. Switching increases with duration of infection, anaemia and other stresses to the parasite population such as partially effective anti-malarial treatment. In *P. falciparum* infections, the developing sexual stages sequester for about 7–10 days in venules and capillaries and particularly in the bone marrow before reentering the circulation as immature stage 5 gametocytes [15]. As a result, peak *P. falciparum* sexual stage densities typically occur approximately 10 days after peak asexual densities [15]. Gametocytes are cleared relatively slowly from the blood so they accumulate with respect to asexual parasites and can predominate in chronic infections. The gametocytes of *P. falciparum* malaria are relatively insensitive to most anti-malarial drugs (with the notable exception of the 8-aminoquinolines) whereas the gametocytes of the other human malaria parasites are considered as drug sensitive as their asexual counterparts [14, 16].

### Synchronicity of the blood stage infection and sequestration

Most natural malaria infections are relatively synchronous so the temporal pattern of parasite density rise in untreated malaria is generally log linear with superimposed oscillations resulting from synchronous schizogony [5, 17] (Fig. 1). The total parasite biomass is the product of the blood volume and the parasite count except in falciparum malaria where, because of sequestration, the peripheral parasite count variably underestimates the total parasite numbers. Sequestration describes the process whereby some 12–18 h after merozoite invasion *P. falciparum* parasitized erythrocytes adhere to vascular endothelium and disappear from the circulation [1]. Once adherent they do not detach until schizont rupture and so the parasites do not reappear in the circulation until the next asexual cycle [18, 19]. This results in a sinusoidal wave form pattern of parasitaemia with sharp rises and falls in parasite density corresponding with schizogony and sequestration, respectively [5] (Fig. 1). In falciparum malaria, large numbers of parasitized erythrocytes accumulate in the placenta and splenic pooling of parasitized erythrocytes may be significant in patients with splenomegaly [2, 20].

### Malaria parasite clearance

Three independent processes contribute to the clearance of malaria parasites from the peripheral blood circulation;

- Host-defence mechanisms
- Anti-malarial drug effects
- Sequestration

In symptomatic malaria, there is usually one dominant normally distributed population of parasite ages [17]. Sometimes “two brood” infections may be observed where two distinct age populations are evident [21]. In uncomplicated malaria, the age distribution of parasites at presentation to medical attention is not random. This is probably because previous cycle schizogony causes a pulse release of pro-inflammatory cytokines which provokes treatment-seeking [22]. Patients with uncomplicated malaria typically present to medical attention with a predominance of young ring stage parasites in the peripheral blood smear indicative of recent schizont rupture [4]. In contrast among patients with severe falciparum malaria the predominant parasite stages in peripheral blood smears appear randomly distributed. Marked fluctuations in parasite density shortly after starting treatment may therefore occur as a natural consequence of the infection itself (Fig. 1). If the majority of parasites in the body are mature schizonts that have not yet ruptured, a sharp rise in parasite count may occur immediately after admission to hospital (these sudden parasitaemia rises also occur in uncomplicated malaria but go unnoticed because frequent parasite counts are seldom made in outpatients) [23–25]. Sudden alarming rises in parasite density were more common following the start of quinine than are now seen after artesunate treatment of severe falciparum malaria. Conversely in a synchronous infection, in which large *P. falciparum* ring stage parasites predominate in the blood smear, there may be a sudden decline in parasite density as these parasites sequester, giving the false impression of an excellent response to the anti-malarial treatment [7].

### *Plasmodium vivax, Plasmodium malariae, Plasmodium ovale, Plasmodium knowlesi*

In all forms of malaria, parasitized erythrocytes can adhere to other erythrocytes (rosetting). *Plasmodium falciparum* and *P. knowlesi*-infected erythrocytes agglutinate with each other at high densities, and *P. vivax* infected erythrocytes can bind to chondroitin sulphate A (a cytoadherence receptor in the placenta), but there is little sequestration in these infections [1]. Except in falciparum malaria all parasite stages of development are seen in peripheral blood smears.

**Fig. 2:**
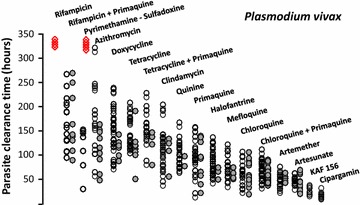
Parasite clearance times in adult Thai patients with vivax malaria after different treatments [28–30]. Parasite counts were determined at ≤6 h intervals on thin films, and at ≤12 h intervals on thick films. The *open circles* are individual asexual parasite clearance times, the *closed circles* are corresponding gametocyte clearance times, and the *red diamonds* denote failure to respond and administration of rescue treatment

Parasite clearance from the blood reflects the stage-specificity and intrinsic potency of the anti-malarial drugs used. The slowest parasite clearance rates are seen following treatment with antibiotics, (e.g. tetracyclines) [26, 27], where the predominant effect is seen the second and subsequent drug-exposed cycles. The most rapid rates are seen following the start of treatment with the artemisinin derivatives and the spiroindolones [23, 28, 29]. The “batting order” of anti-malarial activities (measured in terms of parasite clearance times) in susceptible vivax malaria is similar to that in susceptible falciparum malaria (Fig. 2) with two exceptions; sulfonamides are relatively ineffective in *P. vivax*, and primaquine has only very weak blood stage activity against *P. falciparum* [29, 30].

### *Plasmodium falciparum*

**Fig. 3:**
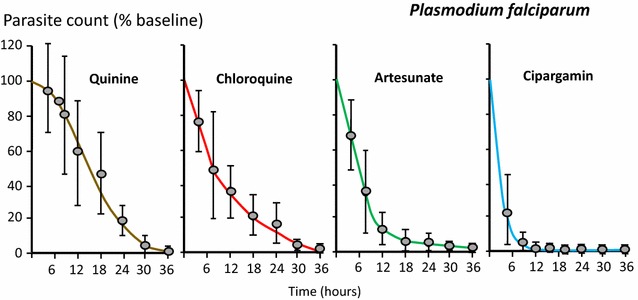
Parasite clearance in acute falciparum malaria. Parasite counts were determined at ≤6 h intervals. These data are taken from studies in severe malaria for choroquine (in fully chloroquine sensitive malaria), quinine and artesunate, and in uncomplicated malaria for cipargamin [28, 33, 42, 56]

**Fig. 4:**
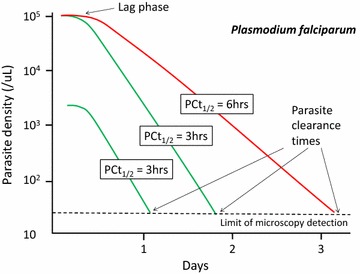
Parasite clearance following the start of anti-malarial drug treatment with an ACT in falciparum malaria. After an initial and variable lag phase, which depends on the stage of parasite development, the decline in parasitaemia is generally log linear [23, 31, 32, 37, 38, 40, 42, 56, 97, 100, 122]. The rate constant of this decline, or its derivative half-life, is the best metric for the assessment of resistance to drugs acting on ring stage parasites-notably artemisinin derivatives [31, 37, 38, 40]. The simpler measure- the proportion of patients who have microscopy detectable parasitaemia on day 3 [100, 101] whilst useful for screening, is heavily dependent on starting parasite density; two infections with the same clearance half-lives (3 h) typically associated with full susceptibility to artemisinin derivative are compared with a 50-fold difference in admission parasitaemia which results in an 18-h difference in parasite clearance time. An artemisinin resistant infection (parasite clearance half-life 6 h) is shown for comparison [38]

Although the parasite density may rise or fall suddenly after starting anti-malarial treatment, in most cases there is a lag phase before parasitaemia falls. Thereafter the decline is log-linear (i.e. clearance is a first order process) [31, 32]. Most anti-malarial drugs have relatively little effect on circulating malaria parasites and so the initial decline in parasite density results both from parasitised red cell sequestration and any ring stage parasite killing and removal [7]. The faster parasite clearance following chloroquine compared with quinine treatment of severe malaria [33] (before chloroquine resistance had emerged) was attributed to a greater effect on ring stage parasites. Parasite clearance is even faster with artemisinin derivatives and the initial lag phase is less evident (Fig. 3) [1, 32]. Rapid clearance results from drug damage to the circulating ring-stage parasites and their subsequent removal predominantly by the spleen [34]. This prevents cytoadherence [35] and the pathological consequences of sequestration, and it largely explains why artesunate reduces mortality substantially in severe falciparum malaria compared with quinine [36]. Artemisinin resistance manifests as loss of ring stage susceptibility and thus slower parasite clearance [37–39]. The slope of the log-linear phase of parasitaemia reduction (or the derived half-life) is particularly useful for assessing resistance to the artemisinins in vivo [31, 32, 37, 38] (Fig. 4), and is the metric which correlates best with heritability (i.e. has the strongest genetic association) [40]. Artemisinin resistance is associated with mutations in the propeller region of the kelch protein [41]. Different mutations confer different levels of resistance (i.e. different mean parasite clearance half-lives: PC_1/2_) [38].

### Measuring parasite clearance

A parasite clearance curve can be constructed from a series of frequent sequential parasite counts, comprising thin film counts at higher densities and thick film counts at lower densities (>50/µL) [31, 32, 38, 42]. Highly sensitive uPCR methods can now quantitate a parasitaemia accurately down to densities of approximately 20/mL. RNA measurement is even more sensitive but as there are changing numbers of transcripts per parasite genome during the asexual life cycle, accurate quantitation of parasitaemia from mRNA measurement is more challenging. As uPCR DNA quantitation is possible at parasitaemias well below the pyrogenic density it is now possible to assess therapeutic responses to anti-malarial drugs in challenge studies without the volunteers becoming ill, and also to follow treated symptomatic infections which later recrudesce and to treat them again before symptoms develop [43–45].

### Mechanisms of parasite clearance

In general, anti-malarial drugs have their greatest activity against mature trophozoites, the most metabolically active stage of asexual parasite development which precedes DNA replication [46, 47]. A possible exception is chloroquine against *P. vivax* [48]. Very young ring stages of *P. falciparum* appear disproportionately sensitive to artemisinins [49]. The damaged and dead parasites in circulating erythrocytes are cleared predominantly by the spleen, as part of its normative function in removing intraerythrocytic particulate matter, although the liver, bone marrow and other lymphoid tissue play an important secondary role in parasitized erythrocyte clearance [34, 50–53]. In falciparum malaria, the sequestered mature trophozoites are killed in situ and then disintegrate slowly. They leave behind erythrocyte membranes adherent to the vascular endothelium, and sometimes trapped malaria pigment, in the once sequestered vessels which is observed in post-mortem brain smears and electron microscopy studies of patients who have died after days of anti-malarial treatment [1, 19, 54, 55]. Clearance of this material is performed by circulating phagocytes (monocytes and polymorphonuclear leukocytes) [56]. At high parasite densities intraleukocytic pigment is observed commonly in blood films, and in severe malaria increased numbers of pigment containing neutrophils (>5%) have prognostic significance [57].

### The spleen

The spleen plays a central role in the control and clearance of intraerythrocytic infections [50]. The spleen’s normal function is to remove senescent red cells and circulating foreign material such as bacteria or cellular debris (often termed “refuse collection” and “policing” activities, respectively) [52, 53]. The structure of the spleen is complex with two overlapping blood circulations—a rapid flow by-pass, called the fast closed circulation, which typically takes 90% of the splenic blood flow (100–300 mL/min in a healthy adult), and a slow-open circulation in which the blood is filtered through narrow inter-endothelial slits. This slow filtration allows the blood elements to be assessed for antibody coating and deformability. Abnormal cells which fail inspection and other particulate material are retained [52, 53, 58]. In malaria, the spleen enlarges rapidly, and is often palpable (i.e. ≥3 times enlarged), and clearance function increases [59–66]. Pathology studies of fatal human malaria which have examined the spleen show marked accumulation of parasitized erythrocytes of all stages [1, 2, 20, 50, 67–71]. Similar findings are reported in primate malaria [72]. Thus, the “activated” spleen retains parasitized red cells (including ring stage infected cells) and it removes parasites and parasitized cells. Splenectomy and splenic dysfunction increase the risk of severe malaria [50, 51, 71], and splenic hypofunction probably contributes to delayed parasite clearance in immunocompromized HIV infected patients receiving anti-malarial treatment [71, 73, 74]. In endemic areas splenomegaly in childhood is used a measure of malaria transmission intensity [4]. There are three processes whereby the spleen can remove malaria parasites.

#### Mechanical filtration

Splenic recognition of reduced erythrocyte deformability and removal of stiff red cells is increased markedly in patients with acute malaria and splenomegaly. Erythrocytes can be made into rigid spherocytes by heating to 51 °C, labelled with a suitable marker, and then used to assess splenic clearance function [75]. The mean half-life (t_½_) for clearance of ^51^Cr-labelled heated red cells in adult Thai patients with acute malaria was 100 min, but this shortened to 20 min by 7–10 days after treatment [63]. In patients presenting with splenomegaly (reflecting longer duration of illness) the t_½_ was 9 min suggesting completely efficient removal of the spherocytic cells each passage through the slow open circulation of the spleen. As *P. falciparum* parasites grow the infected cells becomes more spherical and their deformability is reduced, particularly at the schizont stage [76]. *Plasmodium vivax* does the opposite—as it grows the infected red cell enlarges and becomes more deformable [77]. In severe malaria, the entire red cell population (i.e. uninfected plus infected erythrocytes) becomes stiffer and there is accelerated splenic red cell clearance [78]. This is a major contributor to anaemia. Sequestration in falciparum malaria may have evolved as a mechanism to escape splenic filtration. The spiroindolone cipargamin provides the most rapid parasite clearance yet observed in the treatment of human malaria [28]. This PfATPase 4 inhibitor causes rapid osmotic dysregulation, marked parasite swelling, and increased erythrocyte sphericity. Removal of the whole parasitized erythrocyte by splenic filtration is the likely clearance mechanism [79].

#### Pitting

**Fig. 5:**
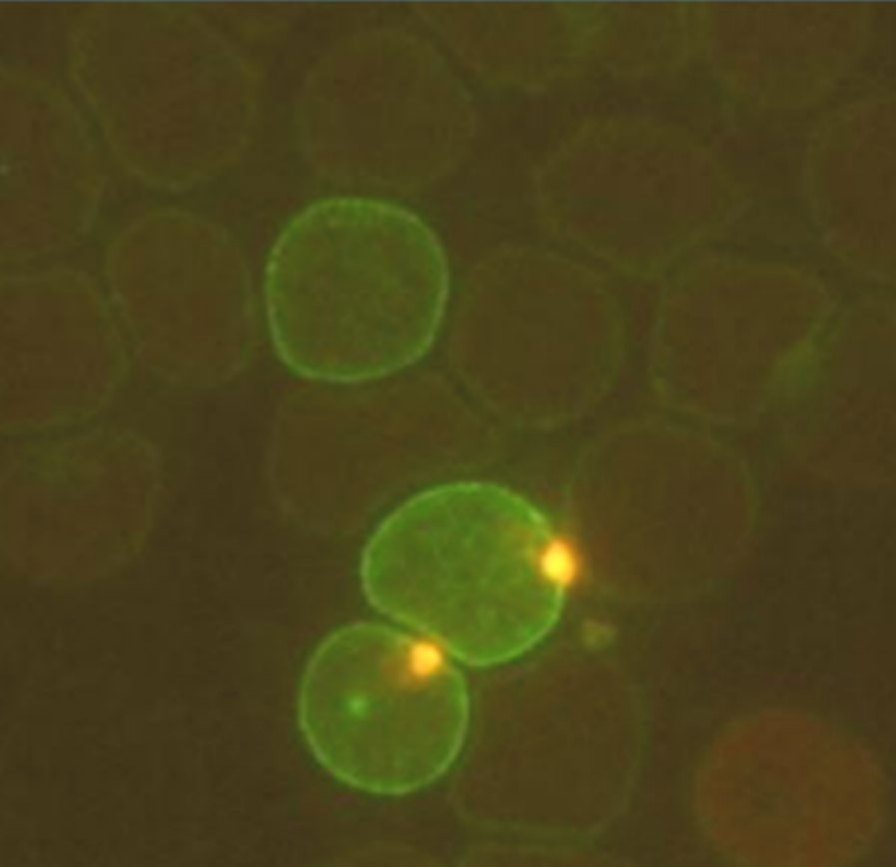
A thin immunofluorescence blood smear showing three red blood cells which stain positive for the *P. falciparum* ring erythrocyte stage antigen. The two lower cells also contain ring stage parasites which stain with acridine orange, the upper cell has no intraerythrocytic parasite indicating that it has already been removed by “pitting”. This is the main mechanism of ring stage parasite clearance in non-immune patients following treatment with artemisinin derivatives [34, 82, 83]

The spleen also removes intraerythrocytic particles such as nuclear remnants (Howell-Jolly bodies), denatured hemoglobin (Heinz bodies) or iron granules (in siderocytes) from intact erythrocytes *without* destroying the cells [52]. The “pitting” capability of the spleen is substantial. Crosby et al. showed that siderocytes could be pitted of their iron granules with a half-life of 80 min in healthy subjects suggesting that pitting rates were close to removal rates for abnormal erythrocytes [80]. Through the same mechanism the spleen also removes damaged circulating intraerythrocytic malaria parasites without destroying the red cells [34, 81–84]. This is the main mechanism of ring stage parasite clearance following treatment with artemisinin derivatives in non-immune patients [82–84]. The pitted “once-infected” erythrocytes can be identified as unparasitized red cells which stain strongly for malaria antigens (Fig. 5). These malaria antigen (RESA) positive parasite-negative red cells (RESA + RBCs) are usually present at low densities before artemisinin treatment, indicating that pitting of young malaria parasites also occurs normally, but their numbers rise in proportion to the decline in parasitaemia after treatment has started. In some patients with falciparum malaria the rise in RESA + RBCs may exceed the decline in parasitaemia indicating that there was splenic retention of ring stage infected erythrocytes before treatment. This process has been elegantly recreated ex vivo by perfusing spleens removed at routine surgery with artesunate-treated parasitized erythrocytes. Sequential Giemsa-stained thin films of the circulating cells in the ex vivo spleen perfusion experiments showed that parasite counts decreased with a half-life of 17–18 min, with an overall clearance time of approximately 120 min [84, 85]. The majority of parasites were retained in the red pulp, as expected from filtration and pitting in the slow-open circulation. The pitting rates by the isolated spleen were comparable to those observed in vivo [83–86].

RESA + RBCs have shortened survival in the circulation [86, 87]. In a study of 14 severe and 6 uncomplicated falciparum malaria patients in Thailand median parasite clearance time was 66 h, and the mean RESA + RBC life was 7.6 days compared with a mean red cell life of 43 days. This accelerated destruction of pitted red cells is an important contributor to post-artesunate haemolysis observed in some hyperparasitaemic non-immune patients following artesunate treatment [87, 88]. Studies in returned French travellers with severe malaria showed that a threshold of 180 million/µL RESA + RBCs identified those patients who would develop delayed haemolysis with 89% sensitivity and 83% specificity [87]. By contrast, in malaria patients who have no spleen dead intraerythrocytic parasites can be seen in the circulation for more than a month following artesunate treatment! [34].

**Fig. 6:**
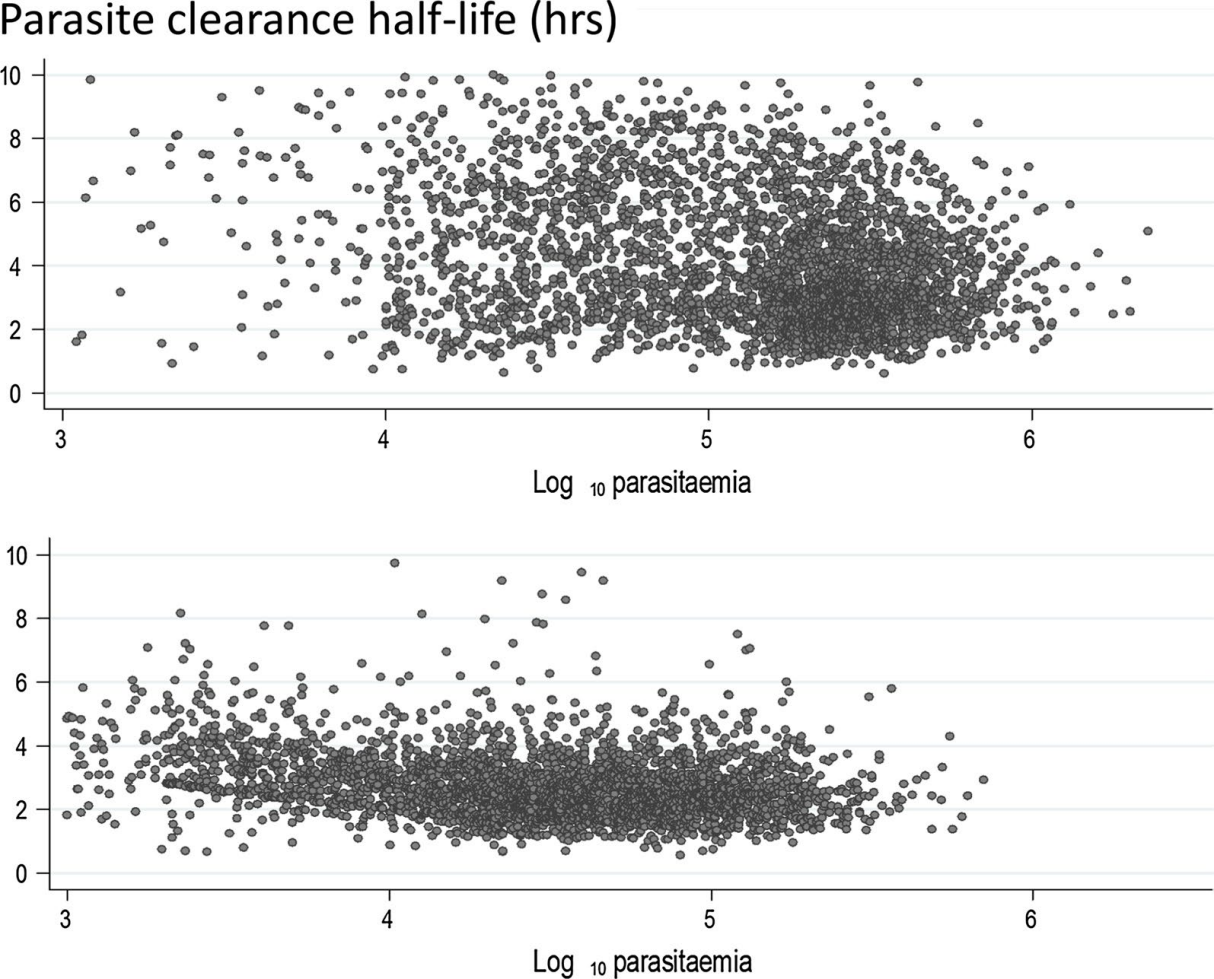
Individual parasite clearance half-lives in relation to presenting parasite density (shown on a log scale per µL) in 6975 patients with acute uncomplicated falciparum malaria treated with an artemisinin derivative (from reference [32]). The *upper panel* shows data from areas unaffected by artemisinin resistance, the *lower panel* shows data from areas where artemisinin resistance is prevalent. There is no evidence for density dependence in parasite clearance rates

It has been suggested the principal determinant of parasite clearance following treatment with artemisinin derivatives is “immunity”, measured as splenic clearance function, and *not* anti-malarial parasiticidal activity [89, 90]. This proposal was based on an earlier PK-PD modelling study of parasite clearance following artemisinin treatment [91]. It was hypothesized that splenic clearance of artemisinin killed parasites is somehow fixed or saturated at 0.26/h, corresponding to a clearance half-life of 2.7 h, and from this it was deduced that dead malaria parasites accumulate in the circulation awaiting splenic removal [89, 90]. Whilst immunity does accelerate parasite clearance this hypothesis, and the deductions based upon it, are very unlikely to be true; all three forms of splenic clearance can exceed this value considerably even in healthy subjects with unprimed spleens [28, 58–60, 62–65, 80, 83–85]. Saturation of splenic clearance function, if it occurred in vivo, should manifest as capacity limitation in the relationship between parasitaemia and parasite clearance following treatment with artemisinin derivatives. This pattern is not observed (Fig. 6).

#### Antibody

Natural antibodies directed against modified band 3 (“senescent antigen”) bind to old erythrocytes resulting in their clearance from the circulation [92]. Membrane-bound anti–band 3 antibodies partially activate complement resulting in red-cell membrane deposition of C3 fragments. The antibody-C3 complex is then readily recognized by phagocyte CR1 complement receptors [93]. This process may be accelerated in malaria infected red cells. The role of immune haemolysis in the pathogenesis of malaria anaemia has been controversial. However it is clear that the threshold for splenic recognition of erythrocyte bound antibody is lowered markedly in malaria, although there is substantial inter-individual variability [64, 65]. Thus, red cells with low antibody coating, which would normally escape clearance, are removed in malaria. As with mechanical clearance, immune clearance usually increases after anti-malarial treatment has started (i.e. as part of the host-defence response to malaria), but unlike mechanical clearance it is *not* increased by splenomegaly. With heavy antibody coating (~8000 molecules per cell) erythrocyte clearance was very rapid in Thai adults with acute malaria—at rates comparable to mechanical clearance [64].

Infusion of malaria hyperimmune serum results in rapid clearance of parasitized erythrocytes. One Thai patient who received 200 mg/kg over 4 h reduced parasitaemia 160-fold within 2 h associated with rapid splenic enlargement [94]. However, at the lower levels of antibody coating more likely to pertain generally in acute malaria clearance half-lives for coated ^51^Cr-labelled autologous erythrocytes halved from approximately 16 to 8 h following anti-malarial treatment [65]. Thus, for parasitized red cells with low antibody coating immune clearance is much slower than either mechanical whole red cell clearance or pitting. As malaria parasites mature they express increasing quantities of antigenic proteins on the infected red cell surface. In falciparum malaria, the predominant surface expressed protein, PfEMP1, is the mediator of cytoadherence [95, 96], so increased antigenicity coincides with sequestration and escape from splenic filtration [1]. In Mali, an area of high malaria transmission, infected erythrocyte opsonization was found to correlate with pitting following artesunate treatment [97]. This may reflect overall augmentation of host-defence mechanisms as antibody mediated clearance would have been expected to result in whole red cell removal.

### Immunity and parasite clearance

Despite enormous research investment and effort immunity to malaria is still poorly understood. In general terms, the acute malaria infection is contained by non-specific host-defence mechanisms including splenic activation and fever (which inhibits schizogony). Later more specific humoral and cellular immunity control and finally eliminate the infection. After weeks of illness in untreated infections parasitaemia is eventually reduced to levels which are tolerated with few or no symptoms. Untreated malaria parasitaemia can persist at low densities for months or years [98]. In malaria-endemic areas, where people are infected frequently, most infections are controlled at densities causing little or no symptoms, so some infections persist for weeks or months and many self- cure [4]. Illness results from infections to which the individual has insufficient immunity to control parasite multiplication [99]. In areas of higher transmission, this is most likely in young children who have had few or no previous infections. In older children and adults rapid mobilization of both non-specific and specific host-defence mechanisms usually results in prompt resolution of the infection—even without anti-malarial treatment. As a result “immunity” complements the effects of anti-malarial drugs, accelerating parasite clearance and augmenting cure rates [100–103]. Failing drugs (i.e. anti-malarials to which resistance has developed) always perform much better in semi-immune patients. Acquired immunity explains why cure rates are always higher in adults and older children in endemic areas and why anti-malarial treatment efficacy assessments in high transmission settings should always include young children [7]. The magnitude of the effect of immunity on parasite clearance can be assessed by comparing parasite clearance rates in drug sensitive infections with similar drug exposure between high transmission and low transmission areas [32, 100], by assessing the effect of age on parasite clearance within an area of moderate or high transmission [101], or directly by correlating parasite clearance rates with malaria antibody titres [104]. In a recent large study the relationship of parasite clearance to titres of antibodies specific to 12 *P. falciparum* sporozoite and blood-stage antigens was assessed. *P. falciparum* antibodies were associated with significantly faster PC_½_ values but the effects were relatively small; maximum shortening <40 min [104]. Immunity also reduces parasite multiplication (e.g. merozoite agglutinating antibodies) but this contributes relatively little to measures of immediate drug effect such as parasite clearance half-lives (PC_1/2_). In the largest assessment to date the effect of age on parasite clearance following treatment with artemisinin derivatives was estimated in a subset of 3208 patients from areas without artemisinin resistance. Young children cleared parasites more slowly than older patients: PC_1/2_ was 11.3% (95% CI 2.6–20.8, p = 0.010) longer in infants aged <1 year and 9.4% (95% CI 3.5–15.7, p = 0.002) longer in children aged 1–4 years compared to older patients. Overall PC_1/2_ values were about 12 min faster in Africa than in Asia, where transmission is generally lower [101].

### Dormancy and parasite clearance

**Fig. 7:**
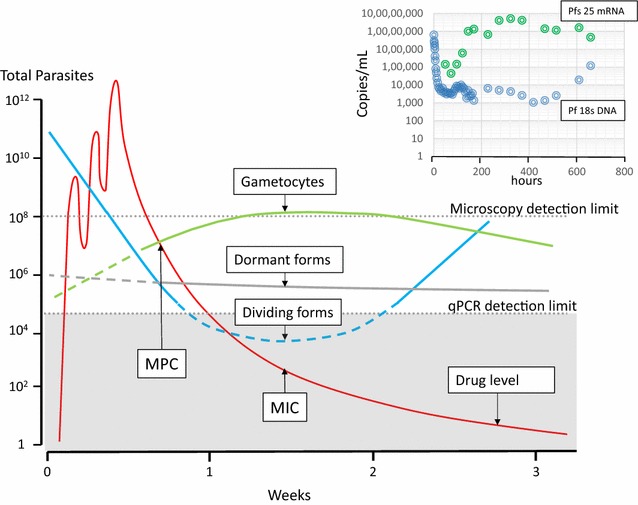
Recrudescent falciparum malaria following administration of a slowly eliminated drug such as mefloquine, showing an example of the changes in total parasite numbers (*blue*) in the body as anti-malarial drug levels (*red*) first rise then fall. As drug levels fall below the minimum parasiticidal concentration (MPC) the rate of parasitaemia declines until it reaches reaching a temporary plateau, at which time the corresponding drug level is a minimum inhibitory concentration (MIC) [23, 117, 124]. Meanwhile levels of any dormant forms remain unchanged, while gametocyte densities rise as stage 5 gametocytes enter the circulation from sequestered sites. All contribute to qPCR parasite DNA measurements. Dormant forms are either cleared or “awaken” to form either asexual or sexual stages. The top right inset shows an individual patient example with female gametocyte specific Pfs 25 mRNA transcript densities shown in* green* and Pf18s DNA shown in* blue* (data from reference [124]). There are multiple mRNA transcripts per cell, but the rising DNA densities at the time of falling transcript numbers clearly indicates recrudescence

There is evidence from both clinical and laboratory studies that asexual blood stage parasites may become temporarily inert or dormant and so survive therapeutic concentrations of anti-malarial drugs. Dormancy is observed particularly following treatment with artemisinin derivatives [6, 105, 106] although it is unclear if the effect is a result of non-lethal cell damage or interference with cell cycling [107] (Fig. 7). It has been suggested that artemisinin resistance reflects an increased propensity for dormancy, although clinical and laboratory studies are more indicative of reduced ring stage artemisinin susceptibility [6, 37, 38, 107–109]. The very high efficacy of ACT outside areas of artemisinin resistance suggests that dormant forms (or more likely these parasites when they wake) do not survive the residual concentrations of partner drugs. In general, dormant parasites are present at densities below the level of microscopy detection, although they may account for some of the “tail” in the parasite clearance curve observed particularly following the treatment of high parasitaemia infections, and they probably contribute significantly to persistent low density uPCR positivity (Fig. 7). The factors associated with dormancy, the metabolic state of the dormant parasites, and their natural history have yet to be characterized fully.

### Gametocytogenesis and parasite clearance

The sexual stages of *P. falciparum* are relatively insensitive to the anti-malarial drugs so they commonly persist after clearance of the asexual stages. Gametocyte densities in falciparum malaria reflect the balance between formation, release from sequestration, and clearance. Dormant forms which “awaken” can also presumably form gametocytes contributing to apparent slow gametocyte clearance. Gametocytes are readily distinguishable by microscopy, but as for dormant parasites, are indistinguishable from asexual parasites by quantitative PCR for DNA. Both therefore contribute to an apparent slow terminal elimination phase of parasite DNA in blood (Fig. 7). Gametocyte clearance also appears to be a first-order process although assessment of the rates of gametocyte clearance is often compromized by their low densities which results in greater errors in determining slopes (Fig. 7). Gametocyte clearance rates derived from microscopy over a few days following drug treatment may have underestimated the true clearance times of drug-unaffected gametocytes [110, 111]. The persistence of low density *P. falciparum* gametocytaemia for weeks after successful treatment of the asexual stage infection is not compatible with current estimates of either drug effects against early stage gametocytes or clearance times. Treatment with primaquine (or methylene blue) leads to rapid *P. falciparum* gametocyte clearance [112]. Gametocyte clearance underestimates drug effects in reducing infectivity. The majority of gametocytes in the circulation are female, yet the most anti-malarials have greater effects on male gametocytes [113]. Sterilization precedes gametocyte clearance. This temporal discrepancy is greatest with the 8-aminoquinolines which sterilize *P. falciparum* infections within hours but the gametocytes take days to clear [16]. Finally, it should be noted that drugs such as antifols and atovaquone may prevent zygote formation in the mosquito without affecting gametocyte clearance [114–116].

### Modelling parasite clearance

Intra-host models of malaria infection have been developed to help characterize anti-malarial drug effects and hopefully guide treatment recommendations and deployment strategies. Anti-malarial drugs are usually modelled to kill parasites by a concentration-dependent process that is first order whilst anti-malarial drugs exceed minimum parasiticidal concentrations (MPC) [23, 89, 90, 108, 117–119]. When anti-malarial drug concentrations fall below the MPC the effect is reduced and the decline in parasitaemia slows. The anti-malarial drug concentration when the parasite multiplication rate (PMR) is one can be termed a minimum inhibitory concentration (MIC) (Fig. 7) [23]. After anti-malarial blood concentrations fall below the MIC, the rise in parasite numbers is determined by the sub-MIC effects on multiplication and the effects of host immunity. Recrudescence occurs when parasitaemias reach densities detectable by microscopy (~50/µL). Stage specificity of anti-malarial drug action, second cycle effects, gametocyte switching, dormancy and increasing immunity can all be incorporated additionally in these PK-PD models.

As the anti-malarial drugs differ in their stage specificities of action [46, 47], the relationship between parasite stage distribution, pattern of drug exposure, parasite killing and clearance is complex. A weakness of many PK-PD models is that parasite killing by anti-malarial drugs is modelled as a single rate constant with a unit of time substantially less than the life span of the cell. A corollary is that for rapidly eliminated anti-malarials such as the artemisinins parasite damage is assumed to stop when drug concentrations decline. Thus, if a drug exceeded parasiticidal concentrations for 8 h (e.g. artemisinins) each day and this resulted in a 10,000-fold reduction in parasite density per asexual cycle, then it could be reasoned that ensuring the drug was present continuously at concentrations above the MPC would result in a 10^12^ fold reduction per day (i.e. 10^4^ × 10^4^ × 10^4^). PK-PD modelling based on this assumption and saturated splenic clearance has concluded that giving artemisinin derivatives twice daily rather than once daily will “dramatically enhance and restore drug effectiveness” particularly in the management of artemisinin resistant falciparum malaria [89, 90, 108]. Clinical studies indicate that is untrue, presumably because single exposures provide near maximum effects, and the more mature sequestered stages remain susceptible in artemisinin resistance [102, 103, 120, 121]. Malaria parasites only need to be killed once in each generation. Current models of the time course of parasite killing may be oversimplifications. Another potential weakness is that parasite killing has been considered equivalent to or greater than parasite removal (mainly by the spleen), whereas it is likely that for drugs acting on ring stages (notably the PfATPase 4 inhibitors) drug affected viable parasites are removed, so splenic clearance rates may exceed the rates of killing of circulating parasites. For drugs which act on ring stage parasites the parasite clearance rate (or derived half-life) is currently the best in vivo measure of drug effect [31, 32, 37, 38, 40, 108, 122].

Current unresolved challenges in pharmacokinetic-pharmacodynamic modelling and anti-malarial dose optimization are how to structure models of parasite clearance, how to characterize the effects of host immunity and parasite “dormancy”, and our incomplete understanding of the behaviour of hypnozoites in *P. vivax* and *P. ovale* infections. Further improvements in parasite quantitation at low densities, particularly the quantitation of low density gametocytaemia, and development of methods which distinguish viable from dead or dying parasites will likely improve model fits, and thus their utility in predicting therapeutic responses. Two experimental approaches provide good characterization of initial anti-malarial responses; the human challenge model (in which “immunity” plays little or no role) [43, 44] and the laboratory model of immunodeficient mice transfused with human blood and infected with *P. falciparum* [123]. Identifying the anti-malarial MIC in an infection requires detailed individual prospective study of pharmacokinetics and sequential quantitation of parasitaemia using both microscopy and uPCR [117, 124]. The MIC is a critical PK-PD variable guiding dose optimization. It provides a method of calibrating in vitro susceptibility data from cultured parasites, and therefore marrying population pharmacokinetic data from different patient groups with susceptibility data from parasites all over the world to inform optimal dosing.

## Conclusions

The spleen plays a central role in the clearance of circulating malaria parasites. Splenic clearance functions increase markedly in acute malaria. Anti-malarial drug treatment damages malaria parasites and either the entire infected erythrocyte is removed or, if the ring stage parasite is affected, the intraerythrocytic parasite may be “pitted” out and the once infected cell is returned to the circulation, where its survival is shortened. Parasite clearance appears to be a first order process. There is no evidence for saturation of the effect. After treatment with artemisinin derivatives some asexual parasites become temporarily dormant, and may regrow after drug exposure. Artemisinin resistance in *P. falciparum* reflects reduced ring stage susceptibility and manifests as slow parasite clearance which is assessed in vivo from the slope of the log-linear phase of parasitaemia reduction. This is commonly measured as a parasite clearance half-life. Sequestered *P. falciparum* parasites are killed in situ by anti-malarial drugs. Pharmacokinetic-pharmacodynamic (PK-PD) modelling of anti-malarial drug effects on parasite clearance has proved useful in predicting therapeutic responses and in dose-optimization.

## Abbreviations

PK-PD: pharmacokinetic-pharmacodynamic
MIC: minimum inhibitory concentration—plasma anti-malarial drug concentration associated with a multiplication rate of 1/asexual cycle
MPC: minimum parasiticidal concentration—lowest plasma anti-malarial drug concentration associated with maximal effects on parasite clearance
RESA + RBCs: ring erythrocyte surface antigen positive parasite negative red cells—once parasitized red cells which have had the malaria parasite “pitted” out by the spleen
uPCR: ultrasensitive polymerase chain reaction detection method for quantitating low density malaria parasitaemia

## References

[1] World Health Organization (2014). Severe malaria. Trop Med Int Health.

[2] Marchiafava E, Bignami A (1894). On summer-autumnal fever.

[3] WHO (2015). Guidelines for the treatment of malaria.

[4] White NJ, Pukrittayakamee S, Hien TT, Faiz MA, Mokuolu OA, Dondorp AM (2014). Malaria. Lancet.

[5] Fairley NH (1947). Sidelights on malaria in man obtained by subinoculation experiments. Trans R Soc Trop Med Hyg.

[6] Cheng Q, Kyle DE, Gatton ML (2012). Artemisinin resistance in *Plasmodium falciparum*: a process linked to dormancy?. Int J Parasitol Drugs Drug Resist..

[7] White NJ (2002). The assessment of antimalarial drug efficacy. Trends Parasitol.

[8] Simpson JA, Aarons L, Collins WE, Jeffery GM, White NJ (2002). Population dynamics of untreated *Plasmodium falciparum* malaria within the adult human host during the expansion phase of the infection. Parasitology.

[9] Dietz K, Raddatz G, Molineaux L (2006). Mathematical model of the first wave of *Plasmodium falciparum* asexual parasitaemia in non-immune and vaccinated individuals. Am J Trop Med Hyg.

[10] Kitchen SF, Boyd MF (1949). Symptomatology: general considerations and falciparum malaria. Malariology.

[11] Kitchen SF, Boyd MF (1949). Vivax malaria. Malariology.

[12] Smith T, Schellenberg JA, Hayes R (1994). Attributable fraction estimates and case definitions for malaria in endemic areas. Stat Med.

[13] Chotivanich K, Udomsangpetch R, Simpson JA, Newton P, Pukrittayakamee S, Looareesuwan S, White NJ (2000). Parasite multiplication potential and the severity of falciparum malaria. J Infect Dis.

[14] Bousema T, Drakeley C (2011). Epidemiology and infectivity of *Plasmodium falciparum* and *Plasmodium vivax* gametocytes in relation to malaria control and elimination. Clin Microbiol Rev.

[15] Thomson D (1911). A research into the production, life and death of crescents in malignant tertian malaria, in treated and untreated cases, by an enumerative method. Ann Trop Med Parasitol.

[16] White NJ, Ashley EA, Recht J, Delves MJ, Ruecker A, Smithuis FM (2014). Assessment of therapeutic responses to gametocytocidal drugs in *Plasmodium falciparum* malaria. Malar J.

[17] White NJ, Chapman D, Watt G (1992). The effects of multiplication and synchronicity on the vascular distribution of parasites in falciparum malaria. Trans R Soc Trop Med Hyg.

[18] Pongponratn E, Riganti M, Punpoowong B, Aikawa M (1991). Microvascular sequestration of parasitised erythrocytes in human falciparum malaria—a pathological study. Am J Trop Med Hyg.

[19] Silamut K, Phu NH, Whitty C, Turner GDH, Louwrier K, Mai NTH (1999). A quantitative analysis of the microvascular sequestration of malaria parasites in the human brain. Am J Path..

[20] Clark HC (1915). The diagnostic value of the placental blood film in aestivo-autumnal malaria. J Exp Med.

[21] Li GQ (1983). [Development state of *Plasmodium falciparum* in the intradermal, peripheral and medullary blood of cerebral malaria patients](in Chinese). Zhonghua Yi Xue Za Zhi..

[22] Dondorp AM, Desakorn V, Pongtavornpinyo W, Sahassananda D, Silamut K, Chotivanich K (2005). Estimation of the total parasite biomass in acute falciparum malaria from plasma PfHRP2. PLoS Med.

[23] White NJ (1997). Assessment of the pharmacodynamic properties of the antimalarial drugs in-vivo. Antimicrob Agents Chemother.

[24] White NJ, Looareesuwan S, Warrell DA, Warrell MJ, Bunnag D, Harinasuta T (1982). Quinine pharmacokinetics and toxicity in cerebral and uncomplicated falciparum malaria. Am J Med.

[25] Gachot B, Houze S, Le Bras J, Charmot G, Bédos JP, Vachon F (1996). Possible prognostic significance of a brief rise in parasitaemia following quinine treatment of severe *Plasmodium falciparum* malaria. Trans R Soc Trop Med Hyg.

[26] Ruiz-Sánchez F, Ruiz-Sánchez A, Naranjo Grande E (1956). The treatment of malaria with tetracycline. Antibiotic Med Clin Therap.

[27] Burkhardt D, Wiesner J, Stoesser N, Ramharter M, Uhlemann AC, Issifou S (2007). Delayed parasite elimination in human infections treated with clindamycin parallels ‘delayed death’ of *Plasmodium falciparum* in vitro. Int J Parasitol.

[28] White NJ, Pukrittayakamee S, Phyo AP, Rueangweerayut R, Nosten F, Jittamala P (2014). Spiroindolone KAE609 for falciparum and vivax malaria. N Engl J Med.

[29] Pukrittayakamee S, Chantra A, Simpson JA, Vanijanonta S, Clemens R, Looareesuwan S (2000). Therapeutic responses to different antimalarial drugs in vivax malaria. Antimicrob Agents Chemother.

[30] Pukrittayakamee S, Clemens R, Chantra A, Nontprasert A, Luknam T, Looareesuwan S (2001). Therapeutic responses to antibacterial drugs in vivax malaria. Trans R Soc Trop Med Hyg.

[31] White NJ (2011). The parasite clearance curve. Malar J.

[32] WWARN Parasite Clearance Study Group (2015). Baseline data of parasite clearance in patients with falciparum malaria treated with an artemisinin derivative: an individual patient data meta-analysis. Malar J.

[33] White NJ, Krishna S, Waller D, Craddock C, Kwiatkowski D, Brewster D (1989). Open comparison of intramuscular chloroquine and quinine in children with severe chloroquine-sensitive falciparum malaria. Lancet.

[34] Chotivanich K, Udomsangpetch R, McGready R, Proux S, Newton P, Pukrittayakamee S (2002). The central role of the spleen in malaria parasite clearance. J Infect Dis.

[35] Udomsangpetch R, Pipitaporn B, Krishna S, Angus B, Pukrittayakamee S, Bates I, Suputtamongkol Y (1996). Antimalarial drugs reduce cytoadherence and rosetting of *Plasmodium falciparum*. J Infect Dis.

[36] Dondorp AM, Fanello CE, Hendriksen ICE, Gomes E, Seni A, Chhaganlal KD (2010). Artesunate versus quinine in the treatment of severe falciparum malaria in African children (AQUAMAT): an open-label, randomised trial. Lancet.

[37] Dondorp AM, Nosten F, Yi P, Das D, Phyo AP, Tarning J (2009). Artemisinin resistance in *Plasmodium falciparum* malaria. N Engl J Med.

[38] Ashley EA, Dhorda M, Fairhurst RM, Amaratunga C, Lim P, Suon S (2014). Tracking resistance to artemisinin collaboration (TRAC). Spread of artemisinin resistance in *Plasmodium falciparum* malaria. N Engl J Med.

[39] Tilley L, Straimer J, Gnädig NF, Ralph SA, Fidock DA (2016). Artemisinin action and resistance in *Plasmodium falciparum*. Trends Parasitol.

[40] Nkhoma SC, Stepniewska K, Nair S, Phyo AP, McGready R, Nosten F (2013). Genetic evaluation of the performance of malaria parasite clearance rate metrics. J Infect Dis.

[41] Ariey F, Witkowski B, Amaratunga C, Beghain J, Langlois AC, Khim N (2014). A molecular marker of artemisinin-resistant *Plasmodium falciparum* malaria. Nature.

[42] Newton P, Angus BJ, Chierakul W, Teerapong P, Dondorp A, Ruangveerayuth R (2003). A randomised comparison of intravenous artesunate or quinine in the treatment of severe falciparum malaria. Clin Infect Dis.

[43] Engwerda CR, Minigo G, Amante FH, McCarthy JS (2012). Experimentally induced blood stage malaria infection as a tool for clinical research. Trends Parasitol.

[44] Marquart L, Baker M, O’Rourke P, McCarthy JS (2015). Evaluating the pharmacodynamic effect of antimalarial drugs in clinical trials by quantitative PCR. Antimicrob Agents Chemother.

[45] Payne RO, Griffin PM, McCarthy JS, Draper SJ (2017). *Plasmodium vivax* controlled human malaria infection—progress and prospects. Trends Parasitol.

[46] ter Kuile F, White NJ, Holloway P, Pasvol G, Krishna S (1993). *Plasmodium falciparum*: in vitro studies of the pharmacodynamic properties of drugs used for the treatment of severe malaria. Exp Parasitol.

[47] Le Manach C, Scheurer C, Sax S, Schleiferböck S, Cabrera DG, Younis Y (2013). Fast in vitro methods to determine the speed of action and the stage-specificity of anti-malarials in *Plasmodium falciparum*. Malar J.

[48] Sharrock WW, Suwanarusk R, Lek-Uthai U, Edstein MD, Kosaisavee V, Travers T (2008). *Plasmodium vivax* trophozoites insensitive to chloroquine. Malar J.

[49] Klonis N, Xie SC, McCaw JM, Crespo-Ortiz MP, Zaloumis SG, Simpson JA (2013). Altered temporal response of malaria parasites determines differential sensitivity to artemisinin. Proc Natl Acad Sci USA.

[50] Garnham PCC (1970). The role of the spleen in protozoal infections with special reference to splenectomy. Acta Trop.

[51] Looareesuwan S, Suntharasamai P, Webster HK, Ho M (1993). Malaria in splenectomised patients: report of four cases and review. Clin Infect Dis.

[52] Crosby WH (1959). Normal functions of the spleen relative to red blood cells: a review. Blood.

[53] Nathan DG (1969). Rubbish in the red cell. N Engl J Med.

[54] MacPherson GG, Warrell MJ, White NJ, Looareesuwan S, Warrell DA (1985). Human cerebral malaria. A quantitative ultrastructural analysis of parasitized erythrocyte sequestration. Am J Pathol.

[55] Pongponratn E, Turner GDH, Day NPJ, Phu NH, Simpson JA, Stepniewska K (2003). An ultrastructural study of the brain in fatal falciparum malaria. Am J Trop Med Hyg.

[56] Day NPJ, Diep PT, Ly PT, Sinh DX, Loc PP, Chuong LV (1996). Clearance kinetics of parasites and pigment-containing leukocytes in severe malaria. Blood.

[57] Phu NH, Day NPJ, Diep TS, Ferguson DJP, White NJ (1995). Intraleukocytic malaria pigment and prognosis in severe malaria. Trans R Soc Trop Med Hyg.

[58] Finch CA, Harker LA, Cook JD (1977). Kinetics of the formed elements of human blood. Blood.

[59] Buffet PA, Safeukui I, Deplaine G, Brousse V, Prendki V, Thellier M (2011). The pathogenesis of *Plasmodium falciparum* malaria in humans: insights from splenic physiology. Blood.

[60] Wyler DJ, Miller LH, Schmidt LH (1977). Spleen function in quartan malaria (due to *Plasmodium inui*): evidence for both protective and suppressive roles in host defense. J Infect Dis.

[61] Smith LP, Hunter KW, Oldfield EC, Strickland GT (1982). Murine malaria: blood clearance and organ sequestration of *Plasmodium yoelii*-infected erythrocytes. Infect Immun.

[62] Wyler DJ (1983). The spleen in malaria. Ciba Found Symp.

[63] Looareesuwan S, Ho M, Wattanagoon Y, White NJ, Warrell DA, Bunnag D (1987). Dynamic alteration in splenic function during acute falciparum malaria. N Engl J Med.

[64] Lee SH, Looareesuwan S, Wattanagoon Y, Ho M, Wuthiekanun V, Vilaiwanna N (1989). Antibody–dependent red cell removal during *P. falciparum* malaria: the clearance of red cells sensitized with an IgG anti-D. Br J Haematol.

[65] Ho M, White NJ, Looareesuwan S, Wattanagoon Y, Lee SH, Walport MJ (1990). Splenic Fc receptor function in host defense and anemia in acute *Plasmodium falciparum* malaria. J Infect Dis.

[66] Kotlyar S, Nteziyaremye J, Olupot-Olupot P, Akech SO, Moore CL, Maitland K (2014). Spleen volume and clinical disease manifestations of severe *Plasmodium falciparum* malaria in African children. Trans R Soc Trop Med Hyg.

[67] Gaskell JF, Millar WL (1920). Studies on malignant malaria in Macedonia. Q J Med.

[68] Spitz S (1946). Pathology of acute falciparum malaria. Mil Med.

[69] Prommano O, Chaisri U, Turner GD, Wilairatana P, Ferguson DJ, Viriyavejakul P (2005). A quantitative ultrastructural study of the liver and the spleen in fatal falciparum malaria. Southeast Asian J Trop Med Public Health.

[70] Milner DA, Lee JJ, Frantzreb C, Whitten RO, Kamiza S, Carr RA (2015). Quantitative assessment of multiorgan sequestration of parasites in fatal pediatric cerebral malaria. J Infect Dis.

[71] Joice R, Frantzreb C, Pradham A, Seydel KB, Kamiza S, Wirth DF (2016). Evidence for spleen dysfunction in malaria-HIV co-infection in a subset of pediatric patients. Mod Pathol.

[72] Schnitzer B, Sodeman TM, Mead ML, Contacos PG (1973). An ultrastructural study of the red pulp of the spleen in malaria. Blood.

[73] Birku Y, Mekonnen E, Björkman A, Wolday D (2002). Delayed clearance of *Plasmodium falciparum* in patients with human immunodeficiency virus co-infection treated with artemisinin. Ethiop Med J.

[74] Muhindo MK, Kakuru A, Jagannathan P, Talisuna A, Osilo E, Orukan F (2014). Early parasite clearance following artemisinin-based combination therapy among Ugandan children with uncomplicated *Plasmodium falciparum* malaria. Malar J..

[75] Crome P, Mollison PL (1964). Splenic destruction of Rh-sensitized, and of heated red cells. Br J Haematol.

[76] Cranston HA, Boylan CW, Carroll GL, Sutera SP, Williamson JR, Gluzman IY (1984). *Plasmodium falciparum* maturation abolishes physiologic red cell deformability. Science.

[77] Suwanarusk R, Cooke BM, Dondorp AM, Silamut K, Sattabongkot J, White NJ (2004). The deformability of red blood cells parasitized by *Plasmodium falciparum* and *P. vivax*. J Infect Dis.

[78] Dondorp AM, Angus BJ, Chotivanich K, Silamut K, Ruangveerayuth R, Hardeman MR (1999). Red cell deformability as a predictor of anemia in severe falciparum malaria. Am J Trop Med Hyg.

[79] Zhang R, Suwanarusk R, Malleret B, Cooke BM, Nosten F, Lau YL (2016). A basis for rapid clearance of circulating ring-stage malaria parasites by the spiroindolone KAE609. J Infect Dis.

[80] Crosby WH (1957). Siderocytes and the spleen. Blood.

[81] Schnitzer B, Sodeman T, Mead ML, Contacos PG (1972). Pitting function of the spleen in malaria: ultrastructural observations. Science.

[82] Angus B, Chotivanich K, Udomsangpetch R, White NJ (1997). In-vivo removal of malaria parasites from red cells without their destruction in acute falciparum malaria. Blood.

[83] Chotivanich K, Udomsangpetch R, Dondorp A, Williams T, Angus B, Simpson JA (2000). The mechanisms of parasite clearance after antimalarial treatment of *Plasmodium falciparum* malaria. J Infect Dis.

[84] Buffet PA, Safeukui I, Milon G, Mercereau-Puijalon O, David PH (2009). Retention of erythrocytes in the spleen: a double-edged process in human malaria. Curr Opin Hematol.

[85] Buffet PA, Milon G, Brousse V, Correas JM, Dousset B, Couvelard A (2006). Ex vivo perfusion of human spleens maintains clearing and processing functions. Blood.

[86] Newton PN, Chotivanich K, Chierakul W, Ruangveerayuth R, Teerapong P, Silamut K (2001). A comparison of the in vivo kinetics of *Plasmodium falciparum* ring-infected erythrocyte surface antigen-positive and—negative erythrocytes. Blood.

[87] Jauréguiberry S, Ndour PA, Roussel C, Ader F, Safeukui I, Nguyen M (2014). Post-artesunate delayed hemolysis is a predictable event related to the lifesaving effect of artemisinins. Blood.

[88] Jauréguiberry S, Thellier M, Ndour PA, Ader F, Roussel C, Sonneville R (2015). Delayed-onset hemolytic anemia in patients with travel-associated severe malaria treated with artesunate, France, 2011–2013. Emerg Infect Dis.

[89] Hastings IM, Kay K, Hodel EM (2015). How robust are malaria parasite clearance rates as indicators of drug effectiveness and resistance?. Antimicrob Agents Chemother.

[90] Kay K, Hodel EM, Hastings IM (2015). Altering antimalarial drug regimens may dramatically enhance and restore drug effectiveness. Antimicrob Agents Chemother.

[91] Gordi T, Xie R, Jusko WJ (2005). Semi-mechanistic pharmacokinetic/pharmacodynamic modelling of the antimalarial effect of artemisinin. Br J Clin Pharmacol.

[92] Bosman GJ, Willekens FL, Werre JM (2005). Erythrocyte aging: a more than superficial resemblance to apoptosis?. Cell Physiol Biochem.

[93] Arese P, Turrini F, Schwarzer E (2005). Band 3/complement-mediated recognition and removal of normally senescent and pathological human erythrocytes. Cell Physiol Biochem.

[94] Sabchareon A, Burnouf T, Ouattara D, Attanath P, Bouharoun-Tayoun H, Chantavanich P (1991). Parasitologic and clinical human response to immunoglobulin administration in falciparum malaria. Am J Trop Med Hyg.

[95] Tembo DL, Nyoni B, Murikoli RV, Mukaka M, Milner DA, Berriman M (2014). Differential PfEMP1 expression is associated with cerebral malaria pathology. PLoS Pathog.

[96] Flick K, Chen Q (2004). var genes, PfEMP1 and the human host. Mol Biochem Parasitol.

[97] Ndour PA, Lopera-Mesa TM, Diakité SA, Chiang S, Mouri O, Roussel C, Jauréguiberry S (2015). *Plasmodium falciparum* clearance is rapid and pitting independent in immune Malian children treated with artesunate for malaria. J Infect Dis.

[98] Ashley EA, White NJ (2014). The duration of *Plasmodium falciparum* infections. Malar J.

[99] Chan JA, Howell KB, Reiling L, Ataide R, Mackintosh CL, Fowkes FJ, Petter M (2012). Targets of antibodies against *Plasmodium falciparum*-infected erythrocytes in malaria immunity. J Clin Invest.

[100] Stepniewska K, Ashley E, Lee SJ, Anstey N, Barnes KI, Binh TQ (2010). In vivo parasitological measures of artemisinin susceptibility. J Infect Dis.

[101] WWARN Artemisinin based Combination Therapy (ACT) Africa Baseline Study Group (2015). Clinical determinants of early parasitological response to ACTs in African patients with uncomplicated falciparum malaria: a literature review and meta-analysis of individual patient data. BMC Med.

[102] WorldWide Antimalarial Resistance Network (WWARN) Lumefantrine PK/PD Study Group (2015). Artemether-lumefantrine treatment of uncomplicated *Plasmodium falciparum* malaria: a systematic review and meta-analysis of day 7 lumefantrine concentrations and therapeutic response using individual patient data. BMC Med.

[103] McIntosh HM, Olliaro P (2000). Artemisinin derivatives for treating uncomplicated malaria. Cochrane Database Syst Rev.

[104] Ataide R, Ashley EA, Powell R, Chan J-A, Malloy M, O’Flaherty K, et al. Host immunity and the assessment of emerging artemisinin resistance: a multinational cohort study. Proc Natl Acad Sci USA. in press. 10.1073/pnas.1615875114 PMC5380044 28289193

[105] Teuscher F, Gatton ML, Chen N, Peters J, Kyle DE, Cheng Q (2010). Artemisinin-induced dormancy in Plasmodium falciparum: duration, recovery rates, and implications in treatment failure. J Infect Dis.

[106] Mok S, Ashley EA, Ferreira PE, Zhu L, Lin Z, Yeo T (2015). Population transcriptomics of human malaria parasites reveals the mechanism of artemisinin resistance. Science.

[107] Witkowski B, Khim N, Chim P, Kim S, Ke S, Kloeung N (2013). Reduced artemisinin susceptibility of *Plasmodium falciparum* ring stages in western Cambodia. Antimicrob Agents Chemother.

[108] Saralamba S, Pan-Ngum W, Maude RJ, Lee SJ, Tarning J, Lindegårdh N (2011). Intrahost modeling of artemisinin resistance in *Plasmodium falciparum*. Proc Natl Acad Sci USA.

[109] Dogovski C, Xie SC, Burgio G, Bridgford J, Mok S, McCaw JM (2015). Targeting the cell stress response of *Plasmodium falciparum* to overcome artemisinin resistance. PLoS Biol.

[110] Bousema T, Okell L, Shekalaghe S, Griffin JT, Omar S, Sawa P (2010). Revisiting the circulation time of *Plasmodium falciparum* gametocytes: molecular detection methods to estimate the duration of gametocyte carriage and the effect of gametocytocidal drugs. Malar J.

[111] WWARN Gametocyte Study Group (2016). Gametocyte carriage in uncomplicated *Plasmodium falciparum* malaria following treatment with artemisinin combination therapy: a systematic review and meta-analysis of individual patient data. BMC Med.

[112] White NJ (2013). Primaquine to prevent transmission of falciparum malaria. Lancet Infect Dis.

[113] Delves MJ, Ruecker A, Straschil U, Lelièvre J, Marques S, López-Barragán MJ (2013). Male and female *Plasmodium falciparum* mature gametocytes show different responses to antimalarial drugs. Antimicrob Agents Chemother.

[114] Rieckmann KH, McNamara JV, Frischer H, Stockert TA, Carson PE, Powell RD (1968). Gametocytocidal and sporontocidal effects of primaquine and of sulfadiazine with pyrimethamine in a chloroquine-resistant strain of *Plasmodium falciparum*. Bull World Health Organ.

[115] Teklehaimanot A, Nguyen-Dinh P, Collins WE, Barber AM, Campbell CC (1985). Evaluation of sporontocidal compounds using *Plasmodium falciparum* gametocytes produced in vitro. Am J Trop Med Hyg.

[116] Fowler RE, Sinden RE, Pudney M (1995). Inhibitory activity of the anti-malarial atovaquone (566C80) against ookinetes, oocysts, and sporozoites of *Plasmodium berghei*. J Parasitol.

[117] White NJ (2013). Pharmacokinetic and pharmacodynamic considerations in antimalarial dose optimization. Antimicrob Agents Chemother.

[118] Simpson JA, Watkins ER, Price RN, Aarons L, Kyle DE, White NJ (2000). Mefloquine pharmacokinetic-pharmacodynamic models: implications for dosing and resistance. Antimicrob Agents Chemother.

[119] Hoshen MB, Heinrich R, Stein WD, Ginsburg H (2000). Mathematical modelling of the within-host dynamics of *Plasmodium falciparum*. Parasitology.

[120] Kremsner PG, Adegnika AA, Hounkpatin AB, Zinsou JF, Taylor TE, Chimalizeni Y (2016). Intramuscular artesunate for severe malaria in African children: a multicenter randomized controlled trial. PLoS Med.

[121] Das D, Tripura R, Phyo AP, Lwin KM, Tarning J, Lee SJ (2013). Effect of high-dose or split-dose artesunate on parasite clearance in artemisinin-resistant falciparum malaria. Clin Infect Dis.

[122] Flegg JA, Guerin PJ, White NJ, Stepniewska K (2011). Standardizing the measurement of parasite clearance in falciparum malaria: the parasite clearance estimator. Malar J.

[123] Jiménez-Díaz MB, Viera S, Fernández-Alvaro E, Angulo-Barturen I (2014). Animal models of efficacy to accelerate drug discovery in malaria. Parasitology.

[124] Hien TT, White NJ, Thuy-Nhien NT, Hoa NT, Thuan PD, Tarning J (2017). Estimation of the in vivo MIC of cipargamin in uncomplicated *Plasmodium falciparum* malaria. Antimicrob Agents Chemother.

