# Erratum to: Malaria parasite clearance

**DOI:** 10.1186/s12936-017-1785-0

**Published:** 2017-05-10

**Authors:** Nicholas J. White

**Affiliations:** Mahidol Oxford Tropical Medicine Research Unit, Faculty of Tropical Medicine, Mahidol University, 420/6 Rajvithi Road, Bangkok, 10400 Thailand

## Erratum to: Malar J (2017) 16:88 DOI 10.1186/s12936-017-1731-1

After publication of the original article [1], it came to the author’s attention that there was an error in the caption of Fig. 6. In the second sentence, the descriptions of the upper and lower panels of the Figure were incorrectly reversed. The correct sentence is as follows:

“The lower panel shows data from areas unaffected by artemisinin resistance, the upper panel shows data from areas where artemisinin resistance is prevalent.”

In addition, there was an error in the ‘Modelling parasite clearance’ section. In the 2nd paragraph, this sentence was incorrect:

“Thus, if a drug exceeded parasiticidal concentrations for 8 h (e.g. artemisinins) each day and this resulted in a 10,000-fold reduction in parasite density per asexual cycle, then it could be reasoned that ensuring the drug was present continuously at concentrations above the MPC would result in a 10^12^ fold reduction per day (i.e. 10^4^ × 10^4^ × 10^4^).”

The sentence should have read as follows:

“Thus, if a drug exceeded parasiticidal concentrations for 8 h (e.g. artemisinins) each day and this resulted in a 10,000-fold reduction in parasite density per asexual cycle (i.e. 100 fold per day), then it could be reasoned that ensuring the drug was present continuously at concentrations above the MPC would result in a 10^6^ fold reduction per day (i.e. 100 × 100 × 100).”
